# Comparison of Microcomb-Based Radio-Frequency Photonic Transversal Signal Processors Implemented with Discrete Components Versus Integrated Chips

**DOI:** 10.3390/mi14091794

**Published:** 2023-09-20

**Authors:** Yang Sun, Jiayang Wu, Yang Li, David J. Moss

**Affiliations:** Optical Sciences Centre, Swinburne University of Technology, Hawthorn, VIC 3122, Australia

**Keywords:** RF photonics, optical microcombs, optical signal processing, photonic integration

## Abstract

RF photonic transversal signal processors, which combine reconfigurable electrical digital signal processing and high-bandwidth photonic processing, provide a powerful solution for achieving adaptive high-speed information processing. Recent progress in optical microcomb technology provides compelling multi-wavelength sources with a compact footprint, yielding a variety of microcomb-based RF photonic transversal signal processors with either discrete or integrated components. Although they operate based on the same principle, the processors in these two forms exhibit distinct performances. This paper presents a comparative investigation of their performances. First, we compare the performances of state-of-the-art processors, focusing on the processing accuracy. Next, we analyze various factors that contribute to the performance differences, including the tap number and imperfect response of experimental components. Finally, we discuss the potential for future improvement. These results provide a comprehensive comparison of microcomb-based RF photonic transversal signal processors implemented using discrete and integrated components and provide insights for their future development.

## 1. Introduction

Driven by the exponential growth of data capacity, there has been a rapid increase in the demand for high-speed information processing. Radio-frequency (RF) photonics, which utilize photonic hardware and technologies to process high-bandwidth RF signals, provide speed advantages over electrical signal processing with intrinsic bandwidth limitations [1]. Additionally, they offer other advantages such as reduced loss and a strong immunity to electromagnetic interference. Although the history of RF photonics can be traced back to the 1970s [2], the advancement of RF photonic signal processing has remained highly active in recent years, along with the rapid developments of integrated optics and high-bandwidth RF devices [1,3,4,5,6,7,8,9]. Particularly, the innovative merging of RF photonics and optical microcombs provides extensive opportunities for real-world applications, such as frequency synthesis [10,11,12,13,14,15], filters [16,17,18,19], and neuromorphic computing [20,21,22,23].

Among the various schemes to implement RF photonic processors, RF photonic transversal signal processors have garnered significant interest due to their exceptional reconfigurability, allowing the realization of diverse processing functions without the need for changing any hardware. Previously, processing functions, such as differentiation [24], integration [25], Hilbert transform [26], arbitrary waveform generation [27], filtering [16,17,18], data transmission [28,29,30,31,32,33], and convolutional processing [20,21], have been successfully demonstrated.

In RF photonic transversal signal processors, a number of wavelength channels are needed to facilitate good reconfigurability and ensure a high processing accuracy. In addition, wide channel spacing between these wavelength channels is necessary to ensure a large operational bandwidth. Recent advances in optical microcomb technology provide a competitive solution to meet these requirements by generating a substantial number of widely spaced wavelengths from a micro-scale resonator, together with the added benefits of a significantly reduced device footprint, power consumption, and complexity [34]. In contrast, conventional multi-wavelength sources, such as discrete laser arrays [35,36], fiber Bragg grating arrays [37,38,39], laser frequency combs generated by electro-optic (EO) modulation [40,41,42], and mode-locked fiber lasers [43,44], suffer from limitations in one form or another, such as a limited number of wavelength channels and insufficient channel spacings.

Early implementations of microcomb-based RF photonic transversal signal processors simply replaced conventional multi-wavelength sources with optical microcombs, while retaining all the other components as discrete devices [1,20]. Although this already yields significant benefits, there is much more to be gained by increasing the level of integration for the entire processing system, particularly with respect to the system size, power consumption, and cost. Recently, several processors comprised entirely of integrated components have also been demonstrated [18,45]. Despite being based on the same operation principle, the processors implemented with discrete and integrated components present different processing performances. Previously, we analyzed the processing accuracy of processors implemented by using discrete components [46]. Given the rapid development of integrated processors, it has become crucial to conduct a comparative analysis of the performances of these two forms. This can provide not only important guidance for their selection and assessment in practical applications, but also insights into state-of-the-art and future developments.

In this paper, we provide a comparative study of the performance of microcomb-based RF photonic transversal signal processors implemented with discrete and integrated components. First, we compare the performance of state-of-the-art processors, focusing on the processing accuracy. Next, we conduct an analysis of multiple factors that induce the performance differences, including the tap number and imperfect response of experimental components. Finally, we discuss the potential for future development. These results provide a comprehensive comparison and valuable perspectives for these processors with high reconfigurability for diverse signal processing applications.

## 2. Microcomb-Based RF Photonic Transversal Signal Processors

**Figure 1:**
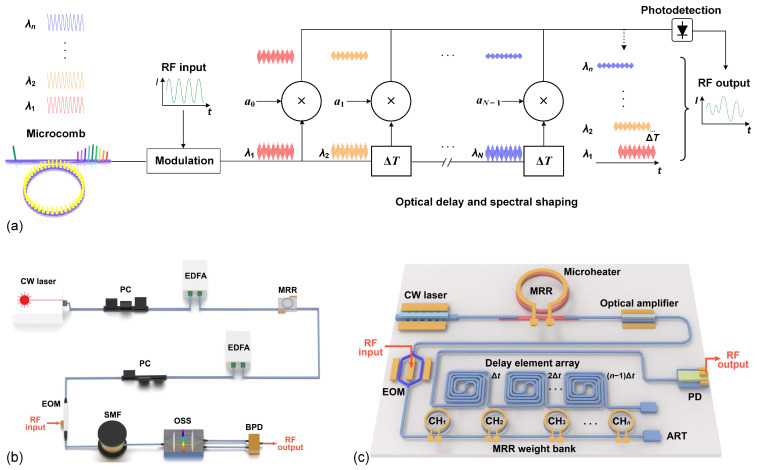
(**a**) Schematic illustration of the operation principle of a microcomb-based RF photonic transversal signal processor. (**b**) Schematic of a microcomb-based RF photonic transversal signal processor implemented by discrete components. (**c**) Schematic of an on-chip microcomb-based RF photonic transversal signal processor implemented with integrated components. EOM: electro-optic modulator. RF: radio frequency. PD: photodetector. CW laser: continuous-wave laser. EDFA: erbium-doped fibre amplifier. PC: polarization controller. MRR: microring resonator. SMF: single-mode fibre. OSS: optical spectral shaper. BPD: balanced photodetector. BPD: balanced photodetector. ART: anti-reflection termination.

RF transversal signal processors are implemented based on the transversal filter structure of digital signal processing, which features a finite impulse response and has been used in applications in a wide range of signal processing functions [1]. Implementing these processors by using RF photonic technology can yield a significantly higher processing bandwidth compared to that of their electronic counterparts [1], and the use of optical microcombs provides a powerful multiwavelength source that is critical for the RF photonic system. Figure 1a illustrates the operation principle of a microcomb-based RF photonic transversal signal processor. The processor employs an optical microcomb as a multiwavelength source, which simultaneously generates numerous wavelength channels as discrete taps. The input RF signal is modulated on each wavelength channel via EO modulation, producing multiple RF replicas. Next, optical spectral shaping is applied to weight these modulated replicas, and a time delay is introduced between adjacent wavelength channels. Finally, the weighted and delayed RF replicas are added together through photodetection to generate the final RF output of the processor. After going through the processing flow in Figure 1a, the output RF signal *s*(*t*) can be given as [1].

(1) $$s(t)=\underset{n=0}{\overset{M-1}{\sum }}a_{n}f(t\;–\;n\Delta T),$$

where *f*(*t*) is the input RF signal, *M* is the tap number, *a_n_* (*n* = 0, 1, 2, …, *M* – 1) is the tap weight of the *n*th tap, and Δ*T* is the time delay between the adjacent wavelength channels. Therefore, the system’s impulse response can be expressed as [1].

(2) $$h(t)=\underset{n=0}{\overset{M-1}{\sum }}a_{n}\delta (t\;–\;n\Delta T),$$

After the Fourier transformation of Equation (2), the spectral transfer function of the processor can be described as

(3) $$H(\omega )=\mathrm{FT}\;[h(t)]=\underset{n=0}{\overset{M-1}{\sum }}a_{n}e^{-j\omega n\Delta T},$$

According to Equations (1)–(3), different processing functions can be realized by appropriately setting the tap coefficients *a_n_* (*n* = 0, 1, 2, …, *M* − 1) without changing the hardware, which allows the high reconfigurability of the processor.

Based on the operation principle in Figure 1a, microcomb-based RF photonic transversal signal processors can be practically implemented into two forms. The first form is illustrated in Figure 1b, where all the components are discrete devices, except for an integrated microring resonator (MRR) used to generate optical microcombs. The second is composed entirely of integrated components, as shown in Figure 1c. To simplify our discussion, we refer to the processors implemented in these two forms as discrete and integrated processors, respectively. Early microcomb-based RF photonic transversal signal processors were developed in the form of discrete processors [1,20], while more recently, some integrated processors have been created [18,45]. Although the operation principle remains consistent across these two forms, their different components result in distinct performances. In the following, we compare their performance in Section 3 and discuss their potential for improvement in Section 4.

## 3. Performance Comparison of Discrete and Integrated Processors

In this section, we compare the performances of the discrete and integrated processors shown in Figure 1b,c, respectively. Although the size, weight, and power consumption (SWaP) of integrated processors are greatly reduced compared with those of the discrete processors, state-of-the-art integrated processors suffer from limited tap numbers due to the restrictions imposed by the integrated components. Currently, integrated processors with only 8 [18] and 12 taps [45] have been demonstrated, whereas discrete processors have been implemented with up to 80 taps [1,46]. The difference in the tap numbers results in a difference in the processing accuracy [46]. In addition, the imperfect response of experimental components also induces processing errors. These mainly include noise from microcombs, the chirp of the EOM, errors in the delay element, and errors in the spectral shaping module [46].

Table 1 summarizes the parameters of the components in the three processors that we investigated, including a discrete processor (Processor 1) and two integrated processors (Processors 2 and 3). There are two integrated processors: one with the same tap number as that in Ref. [18] and another with an increased tap number to demonstrate the potential for improvement. To characterize the errors induced by imperfect experimental components, optical signal-to-noise ratios (OSNRs), chirp parameters (*α*), error of the delay element (*t_v_*), and random tap coefficient errors (RTCEs) are introduced. The OSNR of microcombs is the ratio of the maximum optical signal to the noise power in each of the comb lines [46]. The chirp parameter is used to characterize the chirp of the EOM, which is caused by asymmetry in the electric field overlap at each electrode and can be expressed as [47].

(4) $$\alpha =\frac{\gamma_{1}+\gamma_{2}}{\gamma_{1}\;-\gamma_{2}}$$

where *γ*_1_ and *γ*_2_ are the voltage-to-phase conversion coefficients for the two arms of the modulator. For discrete processors, errors in the delay element are mainly caused by the third-order dispersion of the single-mode fibre, which introduces additional non-uniform time delays between the adjacent wavelength channels. The additional time delay Δ*t* of the *n*th channel is given as [48].

(5) $$\Delta t\;=D_{3}\;l\;\Delta \lambda 2n^{2}$$

where *D*_3_ is the third-order dispersion parameter, *l* is the fibre length, and Δ*λ* is the comb spacing. For integrated processors, errors in the delay element are mainly induced by fabrication imperfections and temperature fluctuations [49]. The errors arising from these sources can be measured via characterizing the RF phase response of each wavelength channel [45,48]. Random tap coefficient errors are the difference between the measured power of comb lines and the ideal tap coefficients. These parameters are all set based on the real processors in Refs. [45,46,47,50,51]. For comparison, we assume that the three processors employ the same microcomb with a comb spacing of 0.4 nm (~50 GHz). We also assume that the time delay between adjacent taps in Equation (3) is ∆*T* = ~33.4 ps to ensure the same operation bandwidth.

Compared with the discrete EOM, the integrated EOM has a relatively high chirp parameter in Table 1, mainly because achieving an accurate bias point and precise electrode placement is more challenging for integrated devices [51]. The lower accuracy for the delay element in the discrete processor is mainly caused by the high-order dispersion of the dispersive medium (e.g., optical fibre), which results in non-uniform time delays between the adjacent wavelength channels [46]. In contrast, in integrated processors, a time delay is introduced by integrated optical delay lines (e.g., Si spiral waveguides), which exhibit a higher accuracy owing to the precise control over the amount of delay achieved by designing a specific length and refractive index profile [52]. The accuracy differences between the integrated and discrete spectral shaping modules are more noticeable. Although the integrated spectral shaping modules have much lower tap numbers, they have lower spectral shaping accuracy compared to that of their discrete counterparts. This is due to the fact that commercial discrete waveshapers based on mature liquid crystal on silicon (LCoS) technology offer much better accuracy for amplitude and phase control, as well as inter-channel synchronization [53].

In our following analysis, three typical signal processing functions, including first-order differentiation (DIF), integration (INT), and Hilbert transform (HT), are taken as examples to compare the accuracy of discrete and integrated processors. The tap numbers required to achieve these processing functions are designed based on our previous work in Ref. [1]. To quantify the comparison of processing accuracy, the root-mean-square error (RMSE) is introduced to compare the deviation between the processors’ outputs and the ideal results, which is expressed as:

(6) $$\mathrm{RMSE}=\sqrt{\underset{i=1}{\overset{k}{\sum }}\frac{(Y_{i}\;–\;y_{i}{)}^{2}}{k}}\;$$

where *k* is the number of sampled points, *Y*_1_, *Y*_2_, …, *Y_n_* are the values of the ideal result, and *y*_1_, *y*_2_, …, *y_n_* are the values of the output of the processors.

**Figure 2:**
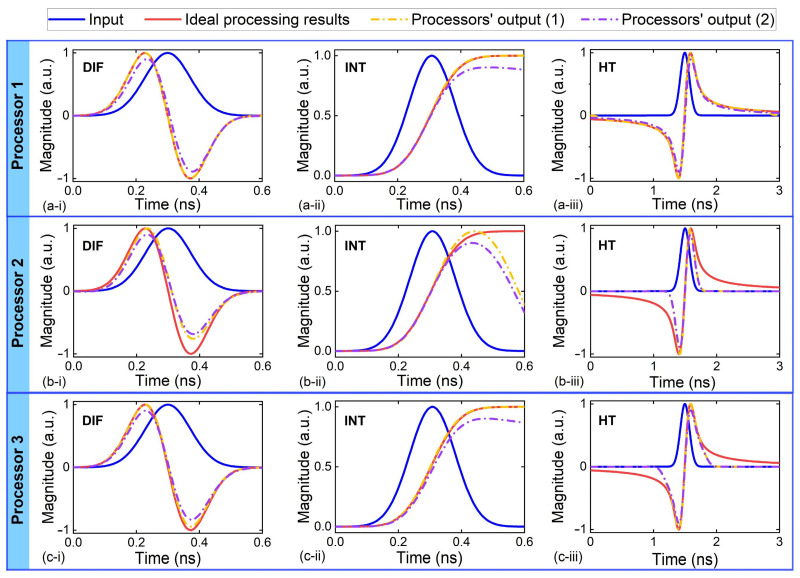
Temporal waveform of Gaussian input pulse and output waveforms from (**a**) Processor 1, (**b**) Processor 2, and (**c**) Processor 3 that performed (**i**) differentiation (DIF), (**ii**) integration (INT), and (**iii**) Hilbert transform (HT). The processors’ outputs with errors induced by (1) only limited tap numbers and (2) both limited tap numbers and experimental errors are shown, together with the ideal processing result for comparison.

Figure 2a–c show the outputs of Processors 1–3 in Table 1 that performed DIF, INT, and HT, respectively. The input RF signal is a Gaussian pulse with a full-width-at-half-maximum (FWHM) of ~0.17 ns. Here, we show the processors’ outputs with errors induced by (1) only limited tap numbers and (2) both limited tap numbers and experimental errors. The ideal processing results are also shown for comparison. Deviations between the processors’ outputs and the ideal results are observed for all three functions, and the deviations become more significant when taking into account the experimental errors.

**Figure 3:**
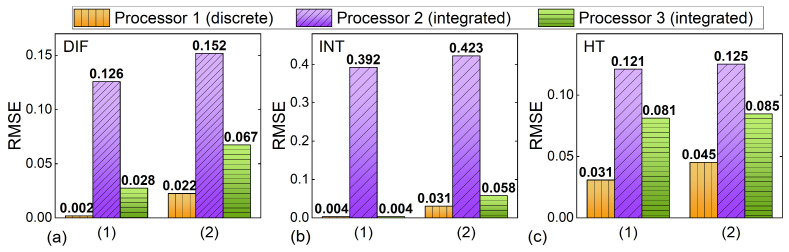
Comparison of root-mean-square errors (RMSEs) of Processors 1–3 that performed (**a**) DIF, (**b**) INT, and (**c**) HT. The RMSEs of processors’ outputs with errors induced by (1) only limited tap numbers and (2) both limited tap numbers and experimental errors are shown.

Figure 3 compares RMSEs of the processors in Figure 2. The higher processing accuracy of the discrete processor compared to that of the integrated processors is reflected by the lower RMSEs of Processor 1 for all three processing functions. In addition, the RMSEs of Processor 3 are lower compared to those of Processor 2, which indicates a higher processing accuracy achieved by increasing the tap number. According to the results in Figure 3, the primary factor that contributes to the degradation of accuracy for integrated processors is the limited tap number, whereas for discrete processors with a sufficiently large tap number, the processing inaccuracy is mainly induced by the imperfect response of the experimental components. We also note that the differences in RMSEs among Processors 1–3 are more prominent for the INT than they are for the other two processing functions, indicating a higher requirement for a greater number of taps to improve the processing accuracy of INT. In addition, experimental errors have a substantial impact on the RMSEs of DIF, whereas their impact on HT is very small.

## 4. Potential for Improvement

**Figure 4:**
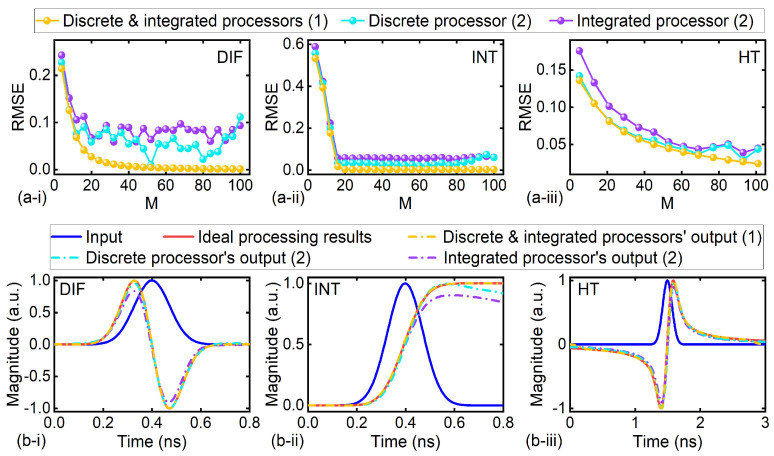
Influence of the tap number and experimental errors on processing accuracy of the discrete and integrated processors. (**a**) RMSEs of processors that performed (**i**) DIF, (**ii**) INT, and (**iii**) HT as a function of tap number M. The RMSEs of processors’ outputs with errors induced by (1) only limited tap numbers and (2) both limited tap numbers and experimental errors are shown. (**b**) Temporal waveform of Gaussian input pulse and output waveforms of discrete and integrated processors that performed (**i**) DIF, (**ii**) INT, and (**iii**) HT. The processors’ outputs with errors induced by (1) limited tap number and (2) both the limited tap number and experimental errors are shown, together with the ideal processing result for comparison.

In this section, we discuss the potential for improvement for both the discrete and integrated processors. In Figure 4a, we quantitively analyze the influence of tap number *M* on the processing accuracy, where the parameters of the input signal and the processor components are kept the same as those in Figure 2. Similar to that in Figure 2 and Figure 3, we show the results with errors induced by (1) only limited tap numbers and (2) both limited tap numbers and experimental errors. It is evident that when assuming no experimental errors, both discrete and integrated processors exhibit the same RMSE values at the same *M*, as they possess identical comb spacing and time delays. When experimental errors are considered, the RMSEs no longer exhibit a monotonic decrease with the tap number *M* as observed when no experimental errors are assumed. This is because some experimental errors, such as shaping errors in both discrete and integrated processors, as well as the increased errors in the time delay induced by higher-order dispersion in discrete processors, increase with *M*. The accumulation of these errors outweighs the reduction in RMSEs caused by increasing *M*, resulting in an overall increase in the RMSEs when *M* exceeds 10 in Figure 4a.

When considering experimental errors, DIF, INT, and HT require tap numbers of 20, 20, and 80, respectively, to achieve an RMSE of ~0.05, respectively. Although this can be easily achieved with the discrete processor, it is challenging for the state-of-the-art integrated processors due to the significantly increased complexity and degraded processing accuracy for *M* ≥ 20. The increased complexity results from the increased numbers of MRRs, micro-heaters, and spiral waveguides in Figure 1c. Although integrated processors have the advantage of monotonically integrating a large number of these building blocks, achieving their precise tuning and control can be challenging, especially when dealing with a large number of taps. On the other hand, fabrication errors, additional losses, and thermal drifts in these building blocks degrade the cooperative operation of different wavelength channels, and the processing errors resulting from these factors increase super linearly with the tap number.

Figure 4b compares the output waveforms of both discrete and integrated processors with the same tap number *M* = 80. Compared to the discrete processor, the experimental errors have more significant influence on the processing accuracy of the integrated processor. Note that we have not taken into account any additional processing errors resulting from factors that may degrade the cooperative operation of different wavelength channels, as discussed earlier. Considering these factors could lead to even larger errors.

To reduce the errors induced by the imperfect response of the experimental components, employing advanced mode-locking approaches [1] to reduce the noise of microcombs could be beneficial for both discrete and integrated processors. For integrated processors, the chirp of silicon EOM can be mitigated by using push–pull configurations, as well as a p-n depletion mode structure [51], and proper methods to calibrate the bias point [45]. The shaping errors of integrated spectral shapers can be alleviated via calibration procedures and gradient descent control [45]. Integrated delay elements introduce additional loss especially when using a waveguide with a high propagation loss, and adiabatic Euler bends can be employed to achieve a reduced loss and low-crosstalk waveguide bends [52]. The use of a wavelength-addressable serial integration scheme can also enable large-scale integration [54]. On the other hand, for discrete processors, there is still room for improving the processing accuracy. Errors in the delay elements induced by higher-order dispersion can be reduced by using programmable phase characteristics of optical spectral shapers (OSSs), and the shaping errors can be minimized through employing feedback control [46].

Environment factors also influence the performance of microcomb-based RF photonic processors. For the MRRs used for generating microcombs, their resonance wavelengths are affected by environment factors such as temperature, thus resulting in the instability of microcombs. The employment of advanced mode-locking technologies has proven to be an effective method to improve the robustness of microcombs, making them less susceptible to environment perturbations [1,3]. Compared to their equivalents implemented using discrete devices, integrated EOMs and spectral shapers are more sensitive to temperature variations. This makes the integrated processors more vulnerable to environmental factors when contrasted with the vulnerability of discrete processors. To minimize the influence of environmental factors in integrated processors, temperature controllers [45] and feedback control [53] have been introduced.

## 5. Conclusions

In summary, we provide a comparative study of the performances of microcomb-based RF photonic transversal signal processors implemented with discrete and integrated components. We first compared the performances of state-of-the-art processors, especially the processing accuracy. Next, analysis of multiple factors that contribute to the performance differences was conducted, including the tap number and imperfect response of the experimental components. Finally, we discussed the potential for future improvement. Our results show that although current integrated processors are attractive in providing a significantly reduced system size, power consumption, and cost, their processing accuracy is not as high as that of the discrete processors. In addition, there is still room for improvement for both the discrete and integrated processors. The results offer valuable insights for microcomb-based RF photonic transversal signal processors with high reconfigurability for diverse applications.

## Figures and Tables

**Table 1:** Comparison of components’ parameters in discrete and integrated processors.

Discrete processor	No.	Tap No.	OSNR of microcombs	Chirp parameter of the EOM	Errors of the delay element	RTCE of the spectral shaping module
	1	*M* = 80 [46]	*OSNR*: 20 dB [46]	*α*: 0.1 [51]	*t_v_*: 4% [46]	*RTCE*: 5% [46]
Integrated processors	No.	Tap No.	OSNR of microcombs	Chirp parameter of the EOM	Error of the delay element	RTCE of the spectral shaping module
	2	*M* = 8 [18]	*OSNR*: 20 dB [46]	*α*: 0.8 [47]	*t_v_*: 3% [45]	*RTCE*: 9% [50]
	No.	Tap No.	OSNR of microcombs	Chirp parameter of the EOM	Error of the delay element	RTCE of the spectral shaping module
	3	*M* = 20	*OSNR*: 20 dB [46]	*α*: 0.8 [47]	*t_v_*: 3% [45]	*RTCE*: 9% [50]

## Data Availability

Not applicable.
